# Targeting naturally occurring epitope variants of hepatitis C virus with high-affinity T-cell receptors

**DOI:** 10.1099/jgv.0.000656

**Published:** 2017-04-01

**Authors:** Huajun Zhang, Jianbing Zhang, Lei Chen, Zhiming Weng, Ye Tian, Haifeng Zhao, Youjia Li, Lin Chen, Zhaoduan Liang, Hongjun Zheng, Wenzhuo Zhao, Shi Zhong, Yi Li

**Affiliations:** ^1^​ State Key Lab of Respiratory Disease, Guangzhou Institutes of Biomedicine and Health, Chinese Academy of Sciences, Guangzhou, PR China; ^2^​ XiangXue Life Sciences Research Center, XiangXue Pharmaceutical Co. Ltd, Guangzhou, PR China; ^†^​ Present address: Wuhan Institute of Virology, Chinese Academy of Sciences, Wuhan, PR China.

**Keywords:** epitope mutation, T cell exhaustion, pHLA, cytotoxic killing, anti-CD3

## Abstract

Hepatitis C virus (HCV) readily establishes chronic infection, which is characterized by failure of virus-specific CD8^+^ T cells. HCV uses epitope mutation and T-cell exhaustion to escape from the host immune response. Previously, we engineered high-affinity T-cell receptors (HATs) targeting human immunodeficiency virus escape mutants. In this study, the affinity of a T-cell receptor specific for the HLA-A2-restricted HCV immunodominant epitope NS3 1406–1415 (KLVALGINAV) was improved from a K_D_ of 6.6 µM to 40 pM. These HATs could also target HCV NS3 naturally occurring variants, including an escape variant vrt1 (KLVVLGINAV), with high affinities. The HATs can be used as high-affinity targeting molecules at the centre of the immune synapse for the HLA-restricted NS3 antigen. By fusing the HAT with a T-cell activation molecule, an anti-CD3 single-chain variable fragment, we constructed a molecule called high-affinity T-cell activation core (HATac), which can redirect functional CTLs possessing any specificity to recognize and kill cells presenting HCV NS3 antigens. This capability was verified with T2 cells loaded with prototype or variant peptides and HepG2 cells expressing the truncated NS3 prototype or variant proteins. The results indicate that HATac targeting the HLA-restricted NS3 antigen may provide a useful tool for circumventing immune escape mutants and T-cell exhaustion caused by HCV infection.

## Introduction

Hepatitis C virus (HCV) is a global public health concern, with about 150 million people in the world chronically infected and more than 350 000 people estimated to die annually from HCV-related liver diseases. After infection, only a minority of patients can resolve spontaneously, whereas 75–85 % of those are unable to clear the virus, thus resulting in chronic infection that potentially leads to severe liver problems, including cirrhosis and liver cancer [1, 2]. The development of direct-acting antiviral (DAA) agents is a breakthrough in the treatment of chronic HCV infection. When used in combination with pegylated IFN and ribavirin, DAAs improve treatment efficacy compared to traditional dual therapy, and extra DAAs make IFN-free combination regimens possible [3]. However, a problem with DAAs is drug resistance [4, 5], and more resistant virus strains may appear as these DAAs are more widely used. Hence, it is desirable to explore novel strategies for treating HCV infection.

Viral-specific T-cell response is believed to play a major role in determining the outcome of HCV infection [6]. Especially in the early stage of infection, the vigour of T-cell response may be a critical determinant of disease resolution and infection control [7]. Chronic HCV infection is characterized by the failure of the viral-specific CD8^+^ T-cell response [8]. Two mechanisms lead to the failure of this response. Accumulating evidence has shed light on the first mechanism, in which HCV can escape the host immune response by generating amino acid mutations in its antigen epitopes [9–13]. For example, Cox *et al*. [9] identified escape mutations in multiple CTL epitopes from seven persistently infected individuals. In another study in which two patients were accidentally infected from a single source but developed either a persistent or resolved infection, an escape mutation was identified from the patient with persistent infection [11]. The escape mutation (named as ‘vrt1’ in this study) occurred in the HLA-A2-restricted immunodominant epitope NS3 1406–1415 (NS3-1406), in which the alanine at the fourth position in the prototype (pt) sequence, KLVALGINAV, was replaced by valine. However, no mutation was observed from the resolved individuals in either study.

The other mechanism for persistent infection is T-cell exhaustion [14], which is a state of T-cell dysfunction that arises during many chronic infections and cancers, whereby antigens can be present for many years [15]. With the expression of inhibitory receptors on their cell surface [16], HCV-specific CD8^+^ T cells can be detected in PBMCs from chronically infected patients but are dysfunctional in proliferation, cytokine production and cytolytic activity [17, 18]. T-cell exhaustion appears to be antigen (HCV) specific, as non-HCV-specific CD8^+^ T cells from chronic HCV patients are fully functional; for example, cytomegalovirus (CMV)-specific CD8^+^ T cells of patients responded well to stimulation of the CMV peptide antigen [18]. Thus, it appears that there is no shortage of CTLs for eliminating viral infected cells; the question is how to promote functionally capable CD8^+^ T cells to target HCV-infected cells and resolve the problem of T-cell exhaustion.

To tackle the two mechanisms of HCV escaping immune control in patients, we propose a way to enhance the capability of T cells to recognize HCV-infected cells, including those with escape mutants, and ultimately cure HCV infection. One of the fundamental elements in T-cell recognition and activation is immunological synapse, which is assembled between a T cell and an antigen-presenting cell with the core molecule pair formation between a T-cell receptor (TCR) and a peptide–HLA (pHLA). Observation of the centre of the immunological synapse with confocal microscopy showed that TCR microclusters, which interact with pHLA molecules, are the fundamental structures associated with T-cell signalling [19]. The optimal agonist signals can only be generated when each TCR microcluster encounters multiple pHLAs [20], and normally, each cell can present hundreds or thousands of pHLAs. This finding indicates that a low number of pHLAs are not sufficient to trigger the T-cell response via a TCR microcluster. The reason may be that the TCR binding to the pHLA is naturally characterized as low affinity with *K*_D_ over 1 µM. We propose that if the binding strength of a TCR toward pHLA is enhanced, fewer copy numbers of pHLAs may be required to activate a T cell. It has been verified that high-affinity TCRs (HATs) [21] can direct a T cell to kill a cancer cell displaying only 50 copies of relevant pHLAs [22], whereas wild-type or low-affinity TCRs fail to produce similar results.

**Fig. 1.**
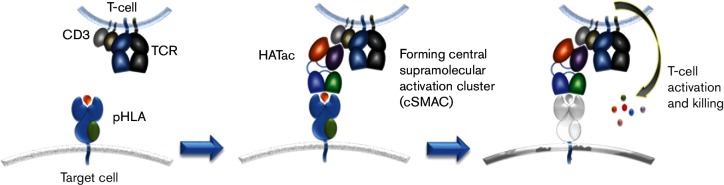
Action of the HATac. The target cell, such as an HCV-infected cell, is bound by HAT fused with an anti-CD3 scFv partner that can bind to T cells possessing any specificity, for example, CMV-specific CD8^+^ T cells for overcoming HCV-specific T-cell exhaustion. Such interaction might form a stable cSMAC, which would result in CMV-specific T-cell activation to kill the HCV-infected target cell.

The idea of using affinity-enhanced TCRs to capture viral escape mutants has been tested with T cells transduced with the gene of HATs recognizing a human immunodeficiency virus (HIV) gag epitope [23]. Here, we investigate whether affinity-enhanced HCV-specific TCRs can be used to eliminate naturally occurring variants of HCV. Instead of using transduced T cells with HATs, we use soluble bi-functional molecules to direct fully functional CTLs possessing any specificity to control HCV escape mutants. Using a TCR against HLA-A2-restricted antigen NS3-1406 [24], we applied phage display libraries to generate three HATs with *K*_D_ ranging from nano- to pico-molar. To investigate whether the HATs could be used to target and lead to cytotoxicity against HCV-infected cells, we constructed a molecule named high-affinity T-cell activation core (HATac), which was a fusion protein with one HAT-containing arm to tightly bind the target cells presenting HLA-restricted epitopes, and another antibody single-chain variable fragment (scFv) arm (aCD3) to bind CD3 at the centre of the immune synapse. In comparison with the natural immune synapse, in which the TCR-rich central supramolecular activation cluster (cSMAC) is formed by the interaction between multiple copies of wild-type TCRs (low affinity) and pHLAs of the target cells [19], a HATac, with HAT to the pHLA, acted as a core to build a new cSMAC and trigger T-cell activation (Fig. 1). The recognition of NS3-1406 and various variants by the HAT was extensively evaluated by surface plasmon resonance (SPR) and cytotoxic assay with peptide-loaded T2 cells and antigen-expressing HepG2 cells. Our results demonstrated that the HATs can recognize NS3-1406 variants with high affinities, and the variation in affinities correlated with the phylogenetic distance. The results also demonstrated that HATacs can redirect CTLs with different specificities to kill cells expressing closely related variant antigens. Thus, the present study presents a good method to tackle naturally occurring T cell epitope variants and T-cell exhaustion, and may provide a novel strategy for viral infection control.

## Results

### Expression of wild-type TCR and affinity maturation

**Fig. 2.**
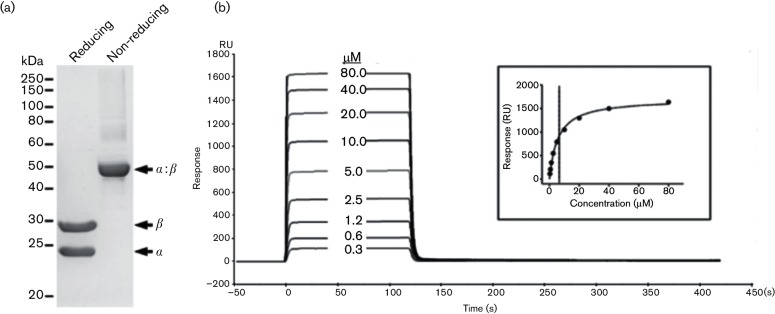
Refolding and purification of wild-type TCR and the affinity measurement. TCR α and β chains were expressed in *Escherichia* *coli* BL21(DE3) as inclusion bodies. Soluble TCR was refolded *in vitro*, dialysed and purified with anion exchange chromatography. Purified TCR was analysed with reducing and non-reducing SDS-PAGE (a). CM5 BIAcore chips were coated with streptavidin using amine coupling, and then biotinylated pt NS3-1406 pHLA A0201 (pHLA-pt-bio) was captured on the active channel. Different concentrations of soluble TCR were injected sequentially through the reference and active channels with multi-cycle kinetics (b). The insert shows the non-linear fit of the Langmuir binding isotherm. The affinity (*K*_D_) was determined to be 6.6 µM using global fitting.

HLA-A2-restricted NS3-1406-specific CD8^+^ T cells and TCRs were generated as previously described by others [24, 25]. To analyse the affinity of the TCR against its cognate antigen, the soluble TCR heterodimer was produced by *in vitro* refolding and purification as described by Boulter *et al*. [26], with yields of 15–20 % (Fig. 2a). Different concentrations of TCRs were injected through BIAcore chips captured with biotinylated pHLA (pHLAbio) using multi-cycle kinetics on BIAcore T200 (Fig. S1, available in the online Supplementary Material). The *K*_D_, the equilibrium dissociation constant between the TCR and the pHLA, was determined to be 6.6 µM using global fitting (Fig. 2b), which was in the typical range found in wild-type TCR against viral antigens [27].

To improve the affinity of the HCV-specific TCR, we constructed phage display libraries containing degenerated complementarity-determining regions (CDRs) of the TCR α and β chains, respectively. We identified two high-affinity mutant sequences from the CDR1α library, one sequence from the CDR1β library, and another two sequences from the CDR3β library (Table 1). As published previously [21], mutant sequences from individual CDR libraries were assembled to achieve combined HAT mutants. The HAT mutant heterodimers were mixtures of different TCR α and β proteins, whereas the biotinylated heterodimer was made through the fusion of a biotin tag to β chains. The successfully prepared HAT heterodimers were immobilized on streptavidin-coated BIAcore chips to determine their binding affinities to pHLA-pt. The single CDR3β HAT mutant had a *K*_D_ of 2 nM, whereas the combined HAT mutants of CDR1α+CDR3β or CDR1α+CDR1β+CDR3β had a *K*_D_ of 140 or 40 pM, respectively (Table 1).

**Table 1.** HATs from phage display selection

TCR	*α* Chain		*β* Chain		*K*_D_ for pHLA-wt
	CDR1	CDR3	CDR1	CDR3	
wt	TSESDYY	AYGEDDKII	MGHDK	ASRRGPYEQY	6.6 µM
HAT-2nM	…….	………	.….	.….SL.LV	2 nM
HAT-140pM	.…E.I	.….….	.….	.….SA.L. .	140 pM
HAT-40pM	…N. .I	………	. .Y. .	.….SL.LV	40 pM

### Recognition of NS3-1406 variants by HATs

We found large amount of HCV sequence data from public HCV databases. To analyse the variants of NS3-1406, we collected 188 full-length HCV polyprotein sequences from GenBank in March 2014. Retrieving NS3-1406 epitope from these polyproteins resulted in 57 variants of NS3-1406, with some variants appearing multiple times. A maximum-likelihood phylogenetic tree was reconstituted for these decapeptides including the most pt-like variant KLVVLGINAV (vrt1), which was reported to be an immune escape mutant [9, 11]. As shown in Fig. S2 and Table 2, the HLA anchor residues at positions 2 and 10 were highly conserved for leucine and valine, respectively, and only one strain (less than 0.6 %) had the second position mutated to phenylalanine. The results of analysing these decapeptides from HCV polyproteins indicated that the mutations mainly occurred at the N-terminal half of the peptide from the third to the fifth residues. However, the first residue of the epitope was mainly glutamine found in 24 strains, followed by lysine in 20 strains, alanine in 13 strains and arginine or threonine in 1 strain. Starting from the C-terminal half of the peptide epitope, position 6 was strictly conserved for glycine and position 9 for alanine. Although position 7 was highly conserved for branched-chain amino acids (valine, leucine or isoleucine), position 8 was almost always asparagine, with less than 2 % of strains having threonine and less than 1 % having histidine. On the basis of natural viral mutation frequency, we prepared pHLA complexes of eight mutant peptides (pHLA-vrt1-8) and the pt peptide (pHLA-pt) for the investigation of HAT capabilities to target naturally occurring variants.

**Table 2.** Recognition of NS3-1406 pHLA-vrts by HATs

pHLA	HAT-40pM			HAT-140pM			HAT-2nM			Sequences*
	k_a_(M^−1^s^−1^)	k_d_ (s^−1^)	*K*_D_ (M)	k_a_ (M^−1^s^−1^)	k_d_(s^−1^)	*K*_D_ (M)	k_a_(M^−1^s^−1^)	k_d_ (s^−1^)	*K*_D_ (M)	
pt	2.7e+05	1.1e−05	4.0e−11	2.9e+05	4.1e−05	1.4e−10	3.2e+05	6.6e−04	2.1e−09	KLVALGINAV
vrt1	3.8e+05	6.3e−04	1.7e−09	4.2e+05	5.2e−04	1.2e−09	4.7e+05	1.1e−01	2.4e−07	KLV**V**LGINAV
vrt2	2.8e+05	2.2e−04	7.8e−10	4.4e+05	3.8e−03	8.6e−09	4.1e+05	2.7e−02	6.6e−08	KL**KS**LG**L**NAV
vrt3	6.6e+05	1.8e−03	2.7e−09	9.9e+05	5.3e−02	5.4e−08	8.5e+05	1.5e−01	1.8e−07	KL**SG**LGINAV
vrt4	2.8e+05	2.8e−03	1.0e−08	1.5e+05	5.9e−02	3.9e−07	2.9e+05	1.0e−01	3.6e−07	**Q**L**TS**LG**L**NAV
vrt5	2.9e+05	6.6e−03	2.3e−08	2.2e+05	1.4e−01	6.4e−07	4.2e+05	4.8e−01	1.1e−06	**Q**L**SS**LG**L**NAV
vrt6	1.0e+04	4.2e−03	4.1e−07	5.1e+03	5.8e−02	1.1e−05	4.1e+03	5.4e−02	1.3e−05	**Q**L**RT**LG**L**NAV
vrt7	4.1e+03	4.1e−03	1.0e−06	1.3e+03	3.7e−02	2.8e−05	1.4e+03	2.9e−02	2.1e−05	**Q**L**RS**LG**L**NAV
vrt8	9.0e+02	4.0e−03	4.4e−06	3.1e+02	2.5e−02	7.8e−05	2.3e+02	1.4e−02	6.0e−05	**A**L**RGM**G**V**NAV

*The varied residues are in bold letters.

**Fig. 3.**
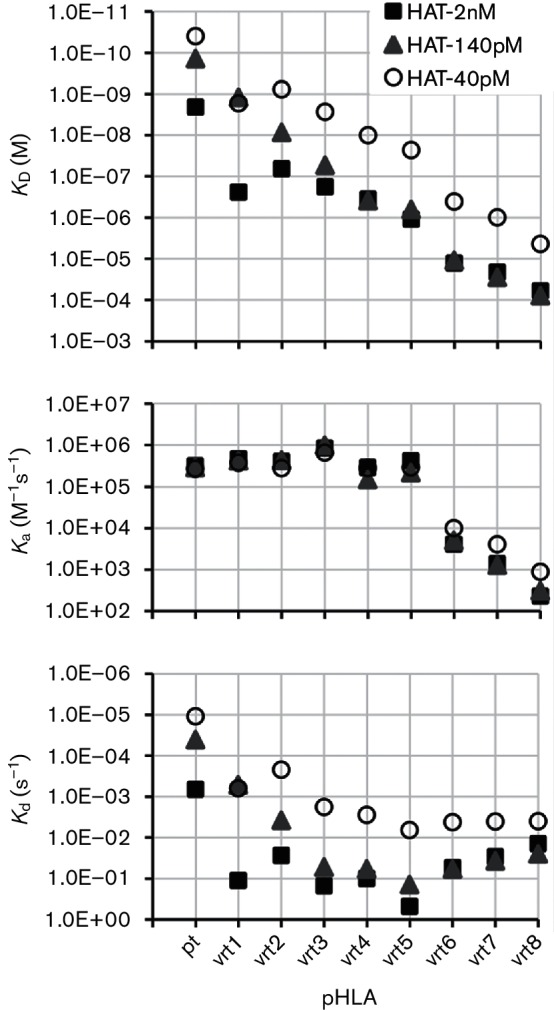
Recognition of pHLA-vrts by HATs. Biotinylated HATs were immobilized on streptavidin-coated CM5 BIAcore chips, and pHLA-vrts were injected at various concentrations with single-cycle kinetics and also run as a negative control in the same setting to get the blank signals. The kinetic constants k_a_, k_d_ and *K*_D_ were determined using BIAcore T200 evaluation software with a 1 : 1 binding model.

The recognition of pHLA-vrts by the three HATs was determined and is shown in Table 2 and Fig. 3. Interestingly, the three HATs recognized pHLA-vrts with decreasing affinities that primarily correlated with the phylogenetic distances of the mutant peptides relative to the pt peptide, with the exceptions of HAT-40pM and HAT-2nM, which recognized pHLA-vrt1 more weakly than pHLA-vrt2. None of the eight pHLA-vrts showed stronger binding with HATs than pHLA-pt, indicating that all three HATs were relatively specific.

For each individual antigen peptide, the three HATs bound pHLA-vrts with similar association rate constants (k_a_), which varied by less than fourfold, but the dissociation rate constants (k_d_) changed by more than 100-fold. For instance, pHLA-vrt1 was bound by HAT-40pM, HAT-140pM and HAT-2nM with a k_a_ of (4.2±0.4)×10^5^ (M^−1^s^−1^), but their *k_d_* values differed by more than 200-fold: 6.3×10^−4^ (M^−1^s^−1^), 5.2×10^−4^ (M^−1^s^−1^) and 1.1×10^−1^ (M^−1^s^−1^), respectively. Data for the binding of each HAT to different pHLAs showed that the *k_a_* values varied within a limited range between 1.5×10^5^ (M^−1^s^−1^) and 9.9×10^5^ (M^−1^s^−1^) for pHLA-pt and pHLA-vrt1-5 and were at least 10 times higher than those for pHLA-vrt6-8, which ranged between 2.3×10^2^ (M^−1^s^−1^) and 1.0×10^4^ (M^−1^s^−1^). However, the *k_d_* data were more complicated. In the case of HAT-40pM, in which the *k_d_* values varied from 1.1×10^−5^ (s^−1^) for pHLA-pt to 6.6×10^−3^ (s^−1^) for pHLA-vrt5, there was almost no change for pHLA-vrt6-8, with *k_d_* values of around (4.1±0.1)×10^−3^ (s^−1^). Moreover, the *k_d_* values of HAT-140pM and HAT-2nM changed from 4.1×10^−5^ (s^−1^) for HAT-140pM binding to pHLA-pt to 4.8×10^−1^ (s^−1^) for HAT-2nM binding to pHLA-vrt5. In contrast, both HATs bound pHLA-vrt6-8 without significant variation in *k_d_* values at around (3.4±2.4)×10^−2^ (s^−1^). In general, the affinities of the binding of the three HATs to pHLA-vrts closely correlated with the number of point mutations in the epitopes, in which more point mutations resulted in weaker binding.

### Cytotoxic activity mediated by HATacs to peptide-loaded T2 cells

**Fig. 4.**
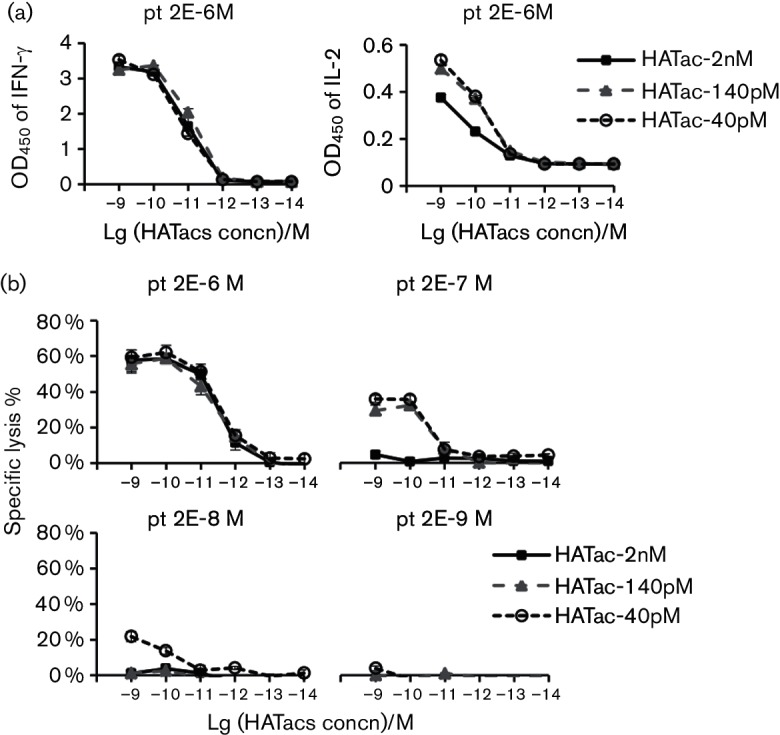
Cytokine release and cytotoxicity assay with T2 cells loaded with pt peptide. (a) T2 cells were loaded with 2×10^−6^ M pt peptides for 2 h and then incubated with expanded CD8^+^ T cells in the presence of HATacs at the indicated concentrations; 20 h later, IFN-γ and IL-2 released in the medium were detected with ELISA. (b) T2 cells were loaded with pt peptide from 2×10^−6^ M to 2×10^−9^ M for 2 h and then incubated with CD8^+^ T cells as above. The specific lysis was determined with a CytoTox 96 Non-Radioactive Cytotoxicity Assay (Promega), which is based on lactatedehydrogenase (LDH) release. *n*=3.

To direct CTLs for killing analysis, HATacs were constructed by fusing aCD3 (UCHT1) to the N-termini of β chains of HAT-2nM, HAT-140pM and HAT-40pM by a GGGGS linker and by refolding with corresponding α chains (Figs 1 and S3). T cells can be activated by HATacs once mixed with cells presenting NS3-1406 peptides with HLA-A2. Activated T cells elicited multiple effector functions, including degranulation and the production of perforin and multiple cytokines. We detected IFN-γ and IL-2 release in the culture media of T2 cells loaded with 2×10^−6^ M pt peptide. Both IFN-γ and IL-2 were released in a HATac concentration-dependent manner (Fig. 4a). There was no difference in IFN-γ release among the three HATacs used, but HATac-2nM elicited less IL-2 than HATac-140pM and HAT-40pM. To investigate the redirected killing by T cells irrespective of their original specificity, we tested the activity of HATacs to direct CD8^+^ T cells to lyse T2 cells loaded with different amounts of NS3-1406 peptide. T2 cells were loaded with serial 10-fold diluted NS3-1406 pt peptide ranging from 2×10^−6^ M to 2×10^−9^ M and then co-cultured with expanded CD8^+^ T cells and the presence of HATacs at various concentrations. As shown in Fig. 4(b), the presence of 2×10^−6^ M pt peptide resulted in no difference in cell lysis between the three HATacs of HATac-2nM, HATac-140pM and HATac-40pM at all concentrations. With the presence of 2×10^−7^ M pt peptide, HATac-2nM did not mediate detectable lysis, whereas HATac-140pM-activated CD8^+^ T cells did lyse the cells to a marginally lower degree than that with HATac-40pM. Moreover, when the pt peptide was diluted to 2×10^−8^ M, only HATac-40pM showed 22 and 14 % specific lysis at the concentrations of 1 and 0.1 nM, respectively, and no significant lysis of T2 cells was detected for all HATacs when the cells were loaded with 2×10^−9^ M pt peptides. These results indicated that the activity to mediate specific lysis was closely related to both the affinity of HATs and the concentration of peptides used for loading the cells.

**Fig. 5.**
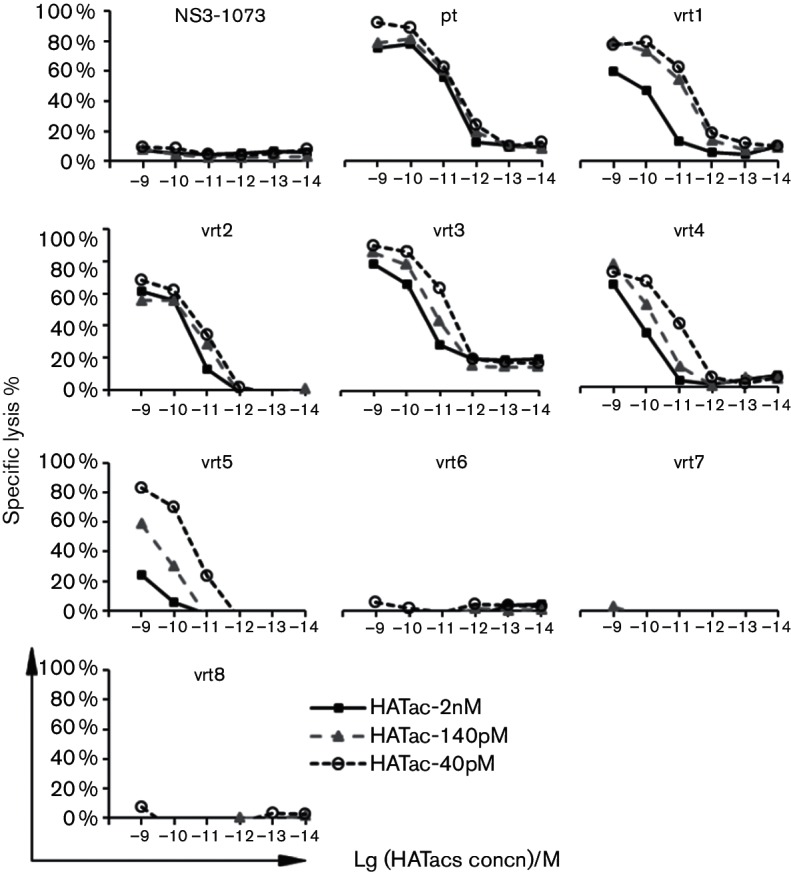
Cytotoxicity assay with T2 cells loaded with mutant peptides. T2 cells were loaded with 2×10^−6^ M of control peptide or NS3-1406 peptides and then analysed as described in Fig. 4. Diagrams show the representative data of two repeated experiments.

When the specific lysis effect on T2 cells was measured for NS3-1406 mutant peptides at the concentration of 2×10^−6^ M, T2 cells loaded with vrt1-5 could be lysed by CD8^+^ T cells activated with HATac-2nM, HATac-140pM or HATac-40pM, but not the cells loaded with vrt6-8 and the negative control peptide NS3-1073 (Fig. 5). In general, for an individual HATac or peptide, the higher the affinity of the TCRs, the greater the percentage of specific lysis to T2 cells loaded with the peptides, indicating that the HATac function of mediating specific lysis was closely related to the affinities of the TCRs.

### Cytotoxic activity mediated by HATacs to epitope-expressing HepG2 cells

**Fig. 6.**
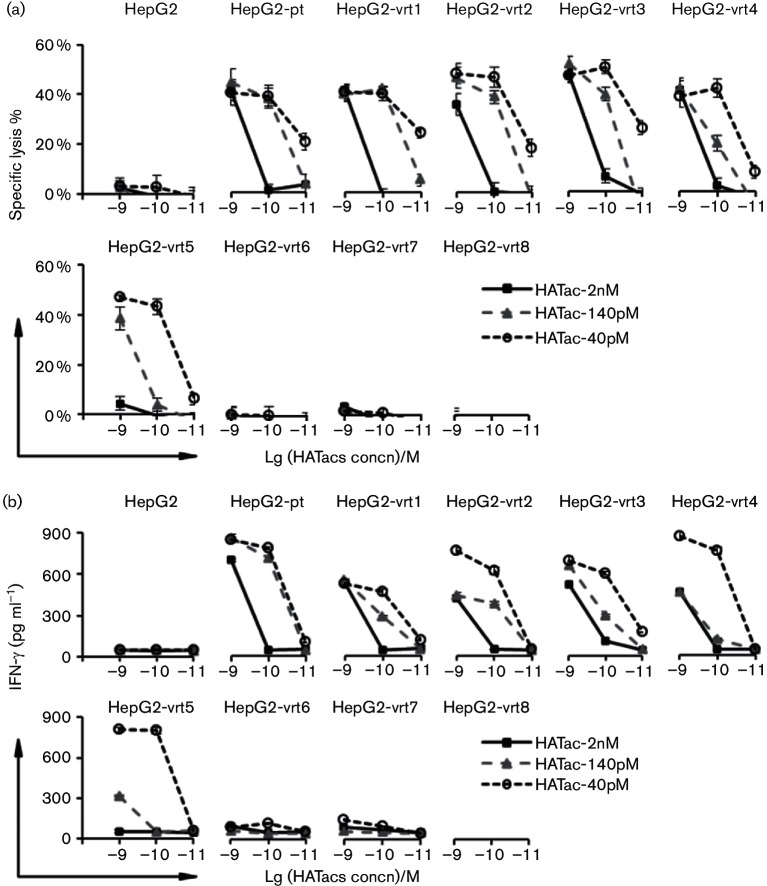
Cytokine release and cytotoxicity assay with HepG2 cell lines expressing NS3-1406 antigens. HepG2 cells were engineered with lentiviral vectors to express truncated NS3-1406 antigens, which were fused with EGFP. The EGFP-positive cells were sorted by FACS. The cells were incubated with CD8^+^ T cells overnight in the presence of HATacs at the indicated concentrations. IFN-γ release (a) and specific lysis (b) were determined as in Fig. 4.

A previous study suggested that HIV-1 immune escape might be controlled by CD8^+^ T cells expressing an affinity-enhanced TCR specific for a pHLA-gag antigen [23]. Similarly to gag-specific TCR-transduced T cells, soluble HATacs could direct lysis to a group of HepG2 cells presenting escape mutants. To establish cell lines that expressed NS3 antigen endogenously and presented NS3-1406 epitopes, we transduced the HLA-A2-positive human hepatoma cell line HepG2 with lentiviral vectors encoding the partial NS3 genes (amino acids 1353–1465) fused with EGFP, and the stably expressing cells were sorted with flow cytometry. These cell lines were co-cultured with CD8^+^ T cells and HATacs at concentrations of 1×10^−9^ M, 1×10^−10^ M and 1×10^−11^ M, respectively. The IFN-γ secretion of CD8^+^ T cells in the media was quantified by ELISA. As shown in Fig. 6(a), IFN-γ was released from the CD8^+^ T cells co-cultured with HepG2-pt and HepG2-vrt1-5 in a manner related to the affinities and doses of HATacs, but no significant IFN-γ release was detected for HepG2-vrt6-8 co-cultured with the CD8^+^ T cells. The specific lysis was also measured separately for each cell line (Fig. 6b). HATac-40pM at 1×10^−11^ M mediated significant lysis to HepG2-pt and HepG2-vrt1-3 and minimum lysis to HepG2-vrt4-5. Significant lysis was observed for HATac-140pM at 1×10^−9^ M and 1×10^−10^ M but not at 1×10^−11^ M, whereas HATac-2nM only mediated significant lysis to HepG2-pt and HepG2-vrt1-4 at 1×10^−9^ M. Consistent with the assay containing peptide-loaded T2 cells, no HATacs mediated specific lysis of HepG2-vrt6-8.

## Discussion

The mechanisms underlying chronic infection with HCV are the generation of viral escape mutants and viral-specific T-cell exhaustion. As recently reviewed by Timm and Walker [28], the CD8^+^ T-cell response shapes viral intra- and inter-host evolution, and in turn, HCV sequence diversity influences the quality of the CD8^+^ T-cell response and thus infection outcome. Epitope mutation was found to lead to a markedly reduced CD8^+^ T cell response. Compared to NS3-1406 pt, the mutation (KLVAMGINAV) was reported to elicit half of the IFN-γ CD8^+^ T-cell response [9], whereas vrt1 (KLVVLGINAV) failed to elicit a peptide-specific response [7, 11]. Redirected by affinity-improved TCR and anti-CD3 scFv, functional non-specific CTL can recognize and kill cells presenting the mutated epitopes or HCV variants, which tackles both viral escape mutations and viral-specific T-cell exhaustion simultaneously.

In addition to escape mutations, HCV has broad range of genotypes. The conserved epitope NS3-1406 varies between the genotypes [29]. Among these variants, an epitope of genotype 1b variant (KLSALGLNAV) showed superior immunogenicity to stimulated CD8^+^ T cells, with relative broad cross-reactivity recognizing several NS3-1406 epitope variants [30], including cross-reactive CD8^+^ T cells against genotype 1a variant (KLVALGINAV), which was used to generate the starting TCR for the current study. In Ziegler *et al.*'s study [30], the epitope of genotype 1a variant (KLVALGINAV) could only stimulate no or little cross-reactive CD8^+^ T cell responses, especially none cross-reactive to genotype 1b variant (KLSALGLNAV). We have also found that, in other in-house studies, different TCRs against the same epitope can show distinct cross-reactivity patterns after affinity enhancement. Based on these studies, it is not difficult to predict that affinity enhancement for TCRs generated with antigen peptides such as genotype 1b variant (KLSALGLNAV) may result in HATs possessing wider cross-reactive attributes.

Previously, we enhanced the affinity of HIV gag-specific TCRs from 150 nM to 400 pM, and the HAT-transduced CD8^+^ T cells recognized and effectively killed HIV escape variants [23]. Here, we generated a series of NS3-1406 antigen-specific HATs [24, 25] with enhanced affinities from the original *K*_D_ of 6.6 µM to 2 nM, 140 pM and even 40 pM. Additionally, we found that these HATs recognized the phylogenetically close mutant antigens, especially vrt1, with high affinity but showed relatively low affinity to the distant mutant antigens.

The HATac molecule is a class of soluble bispecific reagents with two binding moieties, one for pHLA (HAT) and one for CD3 (aCD3 scFv). The dissociation rate constants (k_d_) and association rate constants (k_a_) of the HAT moiety change accordingly with variations of the peptides, but the aCD3 scFv moiety is constant for CD3 with a *K*_D_ of 44 nM, k_a_ of 4.35×10^4^ (M^−1^s^−1^) and k_d_ of 1.61×10^−3^ (s^−1^) (data not shown). Immune-mobilizing monoclonal TCRs against cancer (ImmTACs), which also possess the aCD3 scFv moiety, have been shown to mediate CTL killing, with the efficiency correlated with the strength of the TCR binding to pHLA [22]; however, the relations between k_a_ or k_d_ and CTL killing were not clear in that study. Interestingly, after analysing HATacs with *K*_D_ from 40 pM to 78 µM for a group of pHLA antigens, we found that to effect minimal CTL killing, the k_a_ had to be at least 2.2×10^5^ (M^−1^s^−1^), which was just five times higher than that of the aCD3 scFv binding to CD3. When k_a_ was 2.2×10^5^ (M^−1^s^−1^), even with a k_d_ of 1.4×10^−1^ (s^−1^) and *K_D_* of 640 nM, which indicated about 20 times lower binding efficiency compared to the aCD3 scFv moiety binding to CD3, the HATac-140pM could still mediate CTL killing on cells presenting the vrt5 peptide antigen. In contrast, HATac-40pM, which bound pHLA-vrt6 with higher affinity of *K*_D_ at 410 nM, a k_d_ of 4.2×10^−3^ (s^−1^) and k_a_ of 1.0×10^4^ (M^−1^s^−1^), failed to mediate CTL killing on cells presenting the vrt6 peptide, indicating that a HATac could not activate CTLs if the k_a_ of its HAT for pHLA was weaker than that of the aCD3 scFv binding to CD3. Such a binding mode indicated that, in the most likely case, HATac binding to pHLA is completed before binding to CD3 on T cells.

HATac proteins, especially HATac-40pM, which could recognize several antigen mutants with high affinities and redirect CD8^+^ T cells to kill cells expressing these mutants, could provide a useful tool for eradicating HCV variants with escape mutations. Especially, directed molecular evolution might allow us to further improve the affinity of TCRs against phylogenetically distant mutants and generate new HATacs to capture a broad range of HCV escape variants. Importantly, during chronic infection, HCV-specific CTLs go through the process of exhaustion and fail to eliminate infected cells. HATacs, the fusion proteins of HATs and αCD3 scFv, could redirect non-HCV-specific CTLs to recognize and kill both peptide-loaded T2 cells and HepG2 cells expressing escape mutants. Thus, the HCV antigen-specific T-cell exhaustion can be overcome by harnessing other specific CTLs with the application of HATac.

Pasetto *et al*. [31] proposed examining the possibility of adoptive T-cell immunotherapy using highly functional HCV-reactive T cells. Adoptive transfer of antigen-specific T lymphocytes, genetically engineered with antitumour TCRs, has emerged as an effective therapeutic strategy to combat cancer [32]. Actually, adoptive transfer of virus-specific T cells was used in early-stage clinical trials for haematopoietic cell transplantation recipients to control infections such as those with Epstein–Barr virus [33], CMV [34], adenovirus [35] and polyomavirus JC [36], and showed low toxicity and long-term protection. However, the manufacturing processes to generate such engineered T cells for therapy, including isolation, activation, transduction and expansion, are expensive, labour intensive and time consuming. HATac could bind to target cells through the HAT moiety with high affinity up to pico-molar level and activate T cells through the anti-CD3 moiety, thus circumventing *in vitro* manipulations of T cells. Although early-stage clinical data from a recent study demonstrated potential therapeutic benefits in terms of a durable response and the ultimate breaking of T-cell tolerance via bi-functionally engineered HAT molecules [37], similar reagents have not been developed for virus control (by the time this manuscript had been prepared, similar molecules targeting HIV had been developed by Immunocore). In another study, an enhanced TCR (HAT) was produced for HIV gag-specific epitope SLYNTVATL (SL9), and CD8^+^ T cells transduced with the HAT suppressed HIV infection more effectively than cells transduced with natural SL9-specific TCR [23]. To the best of our knowledge, the present study is the first report of bi-functional T-cell activation molecules for HCV infections.

## Methods

### Cell lines

Cell lines 293T and HepG2 were maintained in DMEM, and cell line T2 was maintained in Iscove's Modified Dulbecco's Medium (IMDM). All culture media were supplemented with 10 % FBS, 100 U ml^−1^ penicillin and 100 g ml^−1^ streptomycin.

After each healthy donor had signed the written consent form, PBMCs were isolated from them with human Lymphoprep reagent (Axis-Shield), and CD8^+^ T cells were isolated from PBMCs with a human CD8^+^ T Cell Isolation Kit (Miltenyi Biotec) according to the manufacturer’s protocol. To expand CD8^+^ T cells, 1×10^6^ CD8^+^ T cells were activated in a six-well plate with Human T-Activator CD3/CD28 Dynabeads (Life Technologies) at a bead-to-cell ratio of 1 : 1 in RPMI 1640 medium supplemented with 10 % FBS, 100 U ml^−1^ penicillin, 100 g ml^−1^ streptomycin and 30 U ml^−1^ recombinant human IL-2. An equal volume of fresh medium was added every 2 or 3 days, and after 7 or 8 days of activation from the start of the culture, CD8^+^ T cells were collected by removing the beads with a magnet.

To engineer HepG2 cells to express the NS3-1406 epitope, we transduced the cells using lentiviral vectors encoding a fusion gene of partial NS3 (amino acids 1353–1465) and the self-cleavage peptide 2A followed by EGFP. The partial NS3 gene was amplified from H/SG-Neo(L+I) plasmid (a kind gift from Professor Charles M. Rice [38]) with the primers NS3-f (5′-gagctagcatgGGCTCCGTCACTGTGT-3′) and NS3-r (5′-gccggatccGCTGAAATCGACTGTCTGAG-3′). To construct the vectors expressing NS3-1406 mutants, a pair of reverse-complement primers (vrt-f and vrt-r) was synthesized covering the mutant sites, and the 5′-end and 3′-end of the partial gene were amplified with NS3-f/vrt-r and vrt-f/NS3-r, respectively, followed by overlapping with NS3-f/NS3-r. The recombinant vectors were co-transfected into 293T cells with packaging vectors pMD2.G, pRSV-REV and pMDLg/pRRE; HepG2 cells were infected with the virus-containing supernatant by centrifugation at 1000 ***g*** for 1 h at 32 °C. The EGFP-positive cells were sorted with a FACSAria II flow cytometer (BD Biosciences).

### Protein expression and inclusion body purification

The pET-28a(+) vector or the modified vector with a biotinylation tag (LNDIFEAQKIEWH) at the C-terminus was used to express the codon-optimized genes coding the extracellular domains of the HLA-A0201 heavy chain (amino acids 25–300), the light chain β2m, the TCR α and β chains, and CD3 γ or ε fusion proteins. All proteins were expressed as inclusion bodies (IBs) in *Escherichia coli* BL21(DE3). To purify IB proteins, cells were collected by centrifugation at 8000 ***g*** for 10 min, and after washing with PBS, the cell pellet was resuspended with BugBuster Master Mix (Novagen, Merck Millipore) and stirred gently at room temperature for 20 min. IBs were spun down at 6000 ***g*** for 15 min and resuspended again with BugBuster Master Mix and stirred for another 5 min. Then, 30 ml of 10-fold diluted BugBuster was added and mixed, followed by centrifugation at 6000 ***g*** for 15 min. IBs were washed twice with 10-fold diluted BugBuster, and after rinsing with deionized water, the IBs were solubilized with the buffer containing 6 M guanidine/HCl, 50 mM Tris/Cl, 100 mM NaCl and 10 mM EDTA for further downstream processes.

### pHLA preparation

The pHLA was refolded essentially as described by Garboczi *et al*. [39], with some modifications. Briefly, 1 mg peptide dissolved in DMSO was added to 40 ml stirred ice-cold refolding buffer (0.1 M Tris/HCl, 0.4 M l-arginine, 2 mM EDTA, 5 mM reduced glutathione, 0.5 mM oxidized glutathione and 0.2 mM PMSF, pH 8.3), followed by 1.2 mg HLA-A2 and 0.8 mg β2m in 1 ml injection buffer (3 M guanidine/HCl, 10 mM sodium acetate, 10 mM EDTA, pH 4.2). HLA-A2 (1.2 mg) was added both 24 and 48 h later, and the refolding mixture was kept at 4 °C with gentle shaking for 3–4 days. At the end of refolding, the mixture was filtered through a 0.44 µm membrane and then dialysed against 10 mM Tris/HCl (pH 8.0) in a 10 kDa cut-off tube at 4 °C for 24 h. The dialysed mixture was loaded onto a QHP column (GE Healthcare) equilibrated with 10 mM Tris/HCl (pH 8.0) and eluted with a linear gradient of NaCl with collection at 1.5 ml per fraction. The fractions with both HLA-A2 and β2m were pooled and concentrated with a 10 kDa Amicon Ultra-15 Centrifugal Filter Unit (Amicon, Merck Millipore). The buffer for the pHLA not requiring biotinylation was changed to PBS in the filter unit, whereas that for the pHLA requiring biotinylation was changed to 10 mM Tris/HCl (pH 8.0). BirA enzyme (Epigen Biotech) was used to biotinylate the tag of pHLA at 20 °C overnight. The reaction mixture was then diluted fivefold with 10 mM Tris/HCl (pH 8.0) and loaded again onto a QHP column to purify the pHLAbio.

### Phage display and HAT selections

The phage display libraries were constructed and screened as described previously [21]. Briefly, v-genes of modified TCR α and β chains encoding a super-stable TCR variable domain (Y. Li, unpublished) were cloned into a phage display vector so that the Vα and Vβ of the TCRs linked with the flexible linker GGGSEGGGSEGGGSEGGGSEGGSGE that was fused at the N-terminal of the phage coat protein III and displayed on the surface of filamentous bacteriophage M13. Then, mutations were introduced into CDR1 and CDR3 of TCR α and β chains by PCR to construct phagemid libraries that were electroporated into TG1 cells. After infection with KM13 helper phage and culturing the cells overnight at 30 °C in a flask with shaking, phage particles were purified by polyethylene glycol precipitation. To select phage particles displaying HATs, a process of bio-panning was performed with pHLAbio-pt captured on streptavidin-coupled Dynabeads (Life Technologies), and then the phage particles were eluted and used to reinfect the TG1 cells. For screening of HATs at the end of three rounds of panning, phage particles from individual colonies were incubated with pHLAbio-pt immobilized on streptavidin-coated 96-well plates, followed by detection with HRP-conjugated anti-M13 mAb (GE Healthcare).

### Preparation of soluble TCR, HAT and HATac

The TCR α- and β-chain IBs were treated with 15 mM DTT at 37 °C for 30–45 min and then rapidly diluted into refolding buffer containing 5 M urea, 0.4 M l-arginine, 100 mM Tris/HCl (pH 8.0), 2 mM EDTA, 3.7 mM cystamine and 6.6 mM β-mercaptoethylamine. The refolding mixture was dialysed overnight against water and then twice against 10 mM Tris/HCl (pH 8.0). Soluble TCR was purified with anion exchange chromatography as above. HATs, whose β chains were fused with the biotin-tag, were further biotinylated and purified similarly as pHLAbio. To purify HATacs, two consecutive rounds of anion exchange chromatography were used, i.e. the fractions from the first round were diluted fivefold with 10 mM Tris/HCl (pH 8.0) and reloaded onto the column, and the fractions from the second elution were pooled, concentrated and further purified with gel filtration. After endotoxin removal, the HATacs were aliquoted and stored at −80 °C. The CD3 was refolded, purified and biotinylated as HAT.

### ELISA

ELISA strips were coated overnight at 4 °C with 100 µl HATacs at 10 µg ml^−1^ in PBS and blocked with PBS containing 5 % skim milk (PBSM) at room temperature for 1 h. Then, 100 µl pHLAbio-pt or biotinylated CD3 (CD3-bio) was added at 10 µg ml^−1^ in PBSM and incubated for 1 h. After washing with PBS containing 0.05 % Tween 20 (PBST), 100 µl HRP-conjugated streptavidin diluted 1 : 5000 in PBSM was added, and the strips were incubated for 20 min. The strips were then washed again with PBST and were developed with Tetramethylbenzidine (TMB) solution. After stopping with 1 M sulfuric acid, the strips were read at 450 nm with an ELISA plate reader. For long-washing ELISA, after incubation with pHLAbio-pt, the strips were washed for the indicated times and immediately frozen at −80 °C until undergoing HRP-conjugated streptavidin incubation.

### Cytotoxic assay

A CytoTox 96 Non-Radioactive Cytotoxicity Assay (Promega), which is based on LDH release, was used to measure cytotoxic lysis. The experiment was set up in 96-well U-bottom plates with RPMI 1640 medium without phenol red supplemented with 5 % FBS in a final volume of 200 µl. Briefly, 4×10^4^ T2 cells were loaded with peptides in 50 µl for 2 h; then, 20 µl HATacs was added at indicated concentrations, followed by 8×10^3^ CD8^+^ T cells in 130 µl volume. Plates were incubated for 20 h at 37 °C in a 5 % CO_2_ incubator and then processed according to the instructions in the kit. The percentage of specific lysis was calculated using the following formula: (experimental well – control well without HATacs)/(target max – target spontaneous)×100 %.

### SPR analysis

The affinity was determined by SPR BIAcore T200 (GE Healthcare). CM5 BIAcore chips were coated with streptavidin using amine coupling, and then biotinylated proteins were captured on the active channel. After blocking both the reference and active channels with 50 mM biotin, unbiotinylated proteins were injected sequentially through the reference and active channels at various concentrations with multi- or single-cycle kinetics. All measurements were performed at 25 °C. BIAcore T200 evaluation software was used to analyse the kinetic constants (k_a_, k_d_ and *K*_D_) with a 1 : 1 binding model.

## Supplementary Data

671 Supplementary File 1
