# Supplementary material for: Targeting naturally occurring epitope variants of hepatitis C virus with high-affinity T-cell receptors
Source: J Gen Virol. 2017 Apr 1;98(3):374–84. doi: 10.1099/jgv.0.000656 (PMC5797947; doi:10.1099/jgv.0.000656)
Supplement: Supplementary File 1 [file jgv-98-374-s001.pdf]

Supplementary information of “Targeting naturally occurring epitope variants of hepatitis C virus with high-affinity T-cell receptors”

Results

Preparation of pHLA. pHLA was prepared by co-refolding HLA-A2 and β2m IBs with peptides and purified with anion exchange chromatography. As illustrated in Fig. S1A, a typical purification chart had three peaks. The first peak was a non-protein peak, followed by soluble β2m peak, and the third consisted of both the heavy chain and the light chain, in which the presence of peptides was confirmed with mass spectrum (data not shown). The fractions in the third peak were pooled and biotinylated. pHLAbio-pt was incubated with Streptavidin magnetic beads. As shown in Fig. S1B, pHLAbio-pt was completely biotinylated, but with some free β2m.

Preparation of HATacs. To generate HATacs, the β chains of HAT-2nM, HAT-140pM and HAT-40pM were fused with the scFv against CD3 (aCD3) by a GGGGS linker, and refolded with corresponding α chains, respectively. HATacs were purified with two rounds of anion exchange chromatography, followed by gel filtration. As wide type TCR, purified HATacs appeared as two bands in reducing SDS-PAGE, and as one band in non-reducing SDS-PAGE (Fig. S3A). We then used ELISA to test the activity of HATacs. Equal amount of HATacs were coated on ELISA strips, and incubated with pHLAbio-pt and CD3-bio to detect the activity of HAT and aCD3 moieties, respectively. As shown in Fig. S3B and S3C, HATac-2nM bound most strongly with CD3-bio, but most weakly with pHLAbio-pt; HATac-140pM bound with CD3-bio and pHLAbio-pt slightly less than HATac-40pM. Since the mutations in HATac-2nM, HATac-140pM and HATac-40pM were very limited in HAT moiety, we reasoned that the difference in CD3-bio binding was due to the variation of refolding efficiency. If the binding activity of pHLAbio-pt was normalized by that of CD3-bio, HATac-140pM and HATac-40pM showed no difference, both significantly higher than

HATac-2nM (Fig. S3D). Each HATac showed no cross activity with the control pHLAbio-NY157 and pHLAbio-NS3(1073).

The pHLAbio-pt binding activity of HATac-140pM and HATac-40pM was inconsistent with their binding affinity. To investigate if it’s related to the quick washing during ELISA, we repeated ELISA with prolonged wash time, that’s, after incubated with pHLAbio-pt, the ELISA strips were washed for 0~8 h and immediately frozen at -80°C until proceeding to HRP-streptavidin incubation. As shown in Fig. S3E, after washed for 8 h, pHLAbio-pt still stayed with HATac-40pM, and was washed away from HATac-140pM after 1 h.

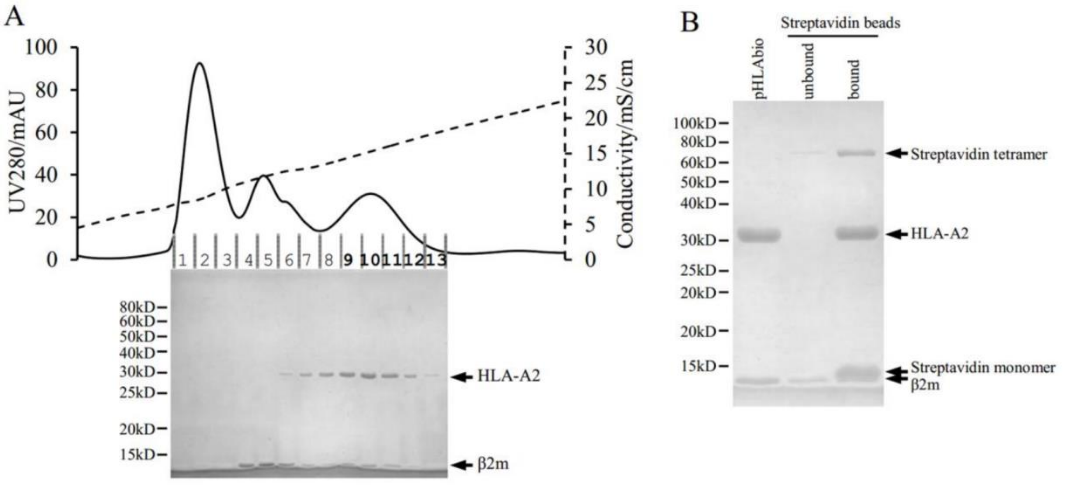

FIG S1 Refolding and purification of pHLA. HLA-A2 and β2m were expressed in *E. coli* BL21(DE3) as inclusion bodies and co-refolded with peptides. After dialysis, soluble pHLA was purified with anion exchange chromatography (A). The fractions 9 to 13 were pooled and biotinylated through the tag on the heavy chain. Biotinylated pHLA (pHLAbio) was incubated with streptavidin beads. The unbound and bound pHLAbio was magnetically separated and analyzed with SDS-PAGE (B).

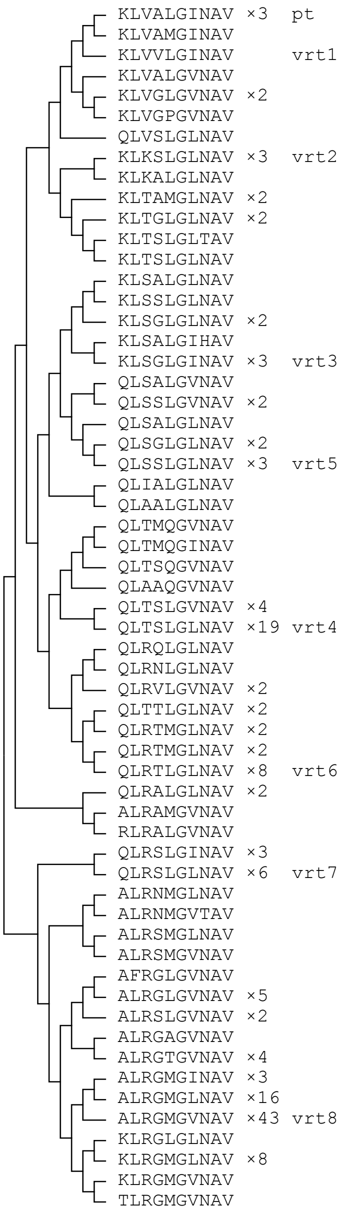

FIG S2 Analysis of HCV NS3-1406 epitope mutations. NS3-1406 epitope sequences were retrieved from 188 full-length HCV polyproteins collected from GenBank around March 2014. The decapeptides were phylogenetically analyzed with MEGA6.0 using the maximum likelihood method based on the Jones-Taylor-Thornton matrix-based model. The numbers indicate the number of times each mutant was observed, and the selected mutants were named “vrt1” to “vrt8.”

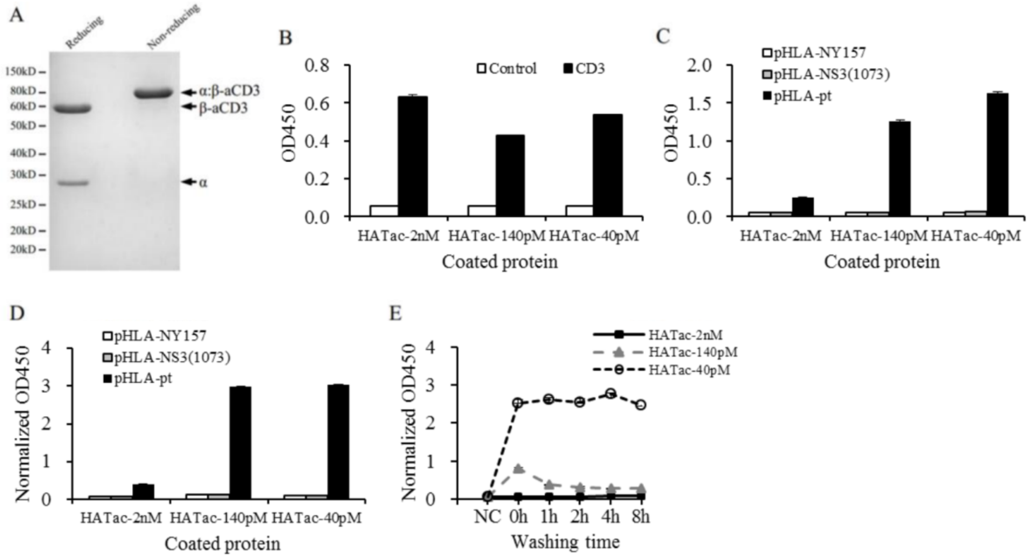

FIG S3 ELISA of HATacs refolded *in vitro*. Equal amount of HATacs were coated on ELISA strips and incubated with CD3-bio (A) and pHLAbio-pt, nonspecific pHLAbio-NY157, and pHLAbio-NS3(1073) (B), followed by streptavidin-HRP. The bindings with pHLAbio were normalized by CD3-bio, respectively (C). (D) Long-washing ELISA. After binding to HATacs, pHLAbio-pt was washed the indicated number of times (E) and immediately frozen at -80°C until streptavidin-HRP incubation. n=2.
